# Social Marketing Strategy to Promote Traditional Thai Medicines during COVID-19: KAP and DoI Two-Step Theory Application Process

**DOI:** 10.3390/ijerph19148416

**Published:** 2022-07-09

**Authors:** Prarawan Senachai, Jakaphun Julsrigival, Raksmey Sann

**Affiliations:** 1 Department of Marketing, Faculty of Business Administration and Accountancy, Khon Kaen University, Khon Kaen 40000, Thailand; prarse@kku.ac.th; 2 Department of Pharmaceutical Science, Faculty of Pharmacy, Chiang Mai University, Chiang Mai 50200, Thailand; jakaphun.jul@cmu.ac.th; 3 Department of Tourism Innovation Management, Faculty of Business Administration and Accountancy, Khon Kaen University, Khon Kaen 40000, Thailand

**Keywords:** traditional Thai medicines, health communication, social marketing, COVID-19, KAP and DoI theory

## Abstract

***Introduction***: Recently, the Thai government has been promoting the innovation of finished forms of traditional Thai medicine (TTM) products (e.g., tablets and capsules). According to the existing literature, most consumers are unaware of the finished forms of TTMs because of conflicting knowledge, information, and communication. Therefore, the consumers have poor perceptions about TTMs and their benefits. ***Purpose***: This qualitative study explores the current perceptions about TTMs and the modes of promotion that are being utilized to develop a strategic communication plan for the finished forms of TTMs. ***Design/methodology/approach***: Utilising thematic analysis, focus groups were conducted with thirty experienced consumers. ***Findings***: Using KAP and DoI theory, the following three themes emerged in this study: (i) the current KAP of Thai consumers toward the finished forms of TTM; (ii) factors influencing the use of finished forms of TTM; and (iii) integrated marketing communication as a promotion strategy to rapidly disseminate knowledge. ***Research limitations/implications***: Given Thailand’s large population, the findings of this study are substantially limited and cannot be generalized. Therefore, the findings herein may not reflect the experiences and opinions of the Thai consumers residing in other regions or the opinions of the entire country. ***Originality/value:*** This study utilises interdisciplinary methods and two-step theory application to explain the current knowledge and perceptions about the finished forms of TTM and develop proper communication and media strategies that can promote the finished forms of traditional Thai medicines, helping to widen their usage significantly.

## 1. Introduction

In 2015, the Ministry of Public Health of Thailand (MoPH) established the following agenda: first, it enabled the use Traditional Thai Medicines (TTM) against minor illnesses, before visiting a medical doctor. This was an open invitation for Thai citizens to start using TTM [1]. Senachai [2] found that the innovation and marketing of TTM products in their finished forms (e.g., tablets and capsules) was being widely promoted by the MoPH. Unfortunately, there is little recognition or awareness about the finished forms of TTM [3].

The literature suggests that there is inadequate knowledge about the finished forms of TTM. Moreover, Thai consumers have obtained inappropriate information about the efficacy of TTM. The communication and media sectors have not yet been inefficient in promoting these medicines. As a result, accurate and appropriate information has not been provided to consumers [2,4]. The consumers believe that the finished forms are undersupplied and only available in hospitals [2,5,6]. Senachai [2] found that such circumstances have taken place because only a few governmental programs have promoted TTM usage. It was found that the media channels responsible for promoting TTM mostly comprised exhibitions and print media, neither of which were able to reach a wide consumer base. There are concerns about the fact that the promotion has targeted only the people living in rural areas. As a result, the population segments belonging to middle and upper classes, and having higher incomes, appear to have been left behind. In essence, the promotion methods being carried out were not properly focused [2]. This was caused by a lack of information or proof supporting TTM’s claims of efficacy and safety [2,7,8]. Later, the promotion stopped, and only a short boom period was experienced [2].

Meanwhile, the private sector promoted the finished forms of TTM as ‘miracle drugs’ (Senachai, 2019, [2] p. 41) capable of curing all illnesses, including cancers [9,10] and paralysis [11]. Some forms of communication were presented to the public without the government’s permission [2,12]. As a result, these forms have been found to violate the drug laws [13]. Consequently, confidence in the finished forms of TTM has been tarnished by inappropriate promotions presented by both sectors [2].

During COVID-19, the use of traditional medicine to cure COVID-19 has received considerable public attention [14]. Traditional medicines, such as Chinese, and Indian medicines, have been used to cure COVID-19 [15]. The Thai government also announced guidelines to support the use of finished forms of TTM. Meanwhile, communication and media played a critical role in fostering the development of the finished forms of TTM [16,17]. However, a few assessments are required to understand this lack of adoption of the finished forms of TTM in Thailand, along with the consumers’ perceptions. The emerging trend in health communication research has advocated the need for participants to place their articulations about health and medicine in the foreground [18,19]. Accordingly, the gap between the ancient knowledge and current information about TTM, provided to the public, and the subsequent communication to promote them, should be significantly investigated. This can help to gain a better understanding of the consumers’ needs in regard to the finished forms of TTM and related healthcare. Thus, communication campaigns can be undertaken to promote the uptake of TTM. Therefore, the main purposes of this study are:

- To understand the current knowledge, attitude, and practice (KAP) with respect to the finished forms of TTM used by Thai consumers.
- To explain the factors influencing the use of finished forms of TTM in Thailand’s marketplace.

In Thai culture, health often includes religion, traditional beliefs [20,21], cuisine, and TTM [21]. Therefore, the Thai lifestyle is based on TTM and should be included to systematically investigate the related health-based research, which has been focused on simple measures of consumption and access. Consequently, there would be a greater comprehension of the necessary guidelines to determine the best strategy promoting the purchase of the finished forms of TTM and specify their usage to achieve the best results. For each population group, accurate information about the finished forms of TTM should be promoted through the proper media channels. This can enhance consumer knowledge, generate positive attitudes, and empower Thai consumers with health issues, enabling them to achieve better quality of health.

## 2. Literature Review

### 2.1. Research Background

Several studies have been conducted to study the attitudes of Thai consumers toward both forms of TTM in Thailand. Ideas have been suggested to develop TTM products and services. This includes the development of proper communication for the promotion of TTM in Thailand’s marketplace [1,2]. However, keywords relevant to “Communication of Traditional Thai Medicine, Communication and Media for the promotion of Traditional Thai Medicine, and Promotion of Traditional Thai Medicine” were scarce in the literature. Meanwhile, Chen and Wang [22] conducted a systematic review to conclude that there are research gaps in understanding the impact of health identity development and addressing privacy concerns. Therefore, studies focusing on the communication and media related to the promotion of the finished forms of TTM, based on customer segmentation in Thailand, have not been conducted. Hypothetically, the tarnished reputation of the finished forms of TTM raises the following question: “*How can the finished forms of Thai Traditional Medicines best be promoted in Thailand’s marketplace by the government?*”

### 2.2. Overview of the Finished Forms of TTM

TTMs have been used in Thailand for almost 800 years and this still continues today [20]. Recently, the Thai government introduced the development of TTMs into the National Strategic Plan (2018–2037) [23]. Thus, in the following 20-year period, TTM will receive great support from the government. However, only one out of five Thai citizens were aware of this policy implementation and had significantly used the finished forms of TTM. This is because only a few programs were being implemented by the Thailand government to promote the usage of finished forms of TTM [2]. According to the literature, the key failures in promoting the finished forms of TTM originate from the biases and bad attitudes of consumers, who have been influenced by Western beliefs. The promotion of the finished forms of TTM, by both sectors, has been inefficient. Furthermore, the content had been misleading and inaccurate claims have been made regarding the pharmaceutical properties of the drugs without the approval of the government [12].

Many studies from Thailand have claimed that the product quality and the packaging design of the finished forms of TTM look cheap and unclean [1,2,7]. These factors reflected poorly on the reliability of the products. In fact, the packaging of the finished products strong influences the consumers’ purchasing decisions, especially among people belonging to the middle-class [2]. Some consumers were not able to correctly identify whether a herbal product was a food, cosmetic, or a drug [24]. This might be because several food advertisements claim to have therapeutic properties. Therefore, most consumers believed in the drug’s efficiency, according to the claim of the advertisements. Nonetheless, the impact of the misleading advertisements on consumers’ perceptions has led the majority of consumers to believe that the products can be beneficial in addressing health problems [2].

Clearly, there are conflicts about knowledge, socio-structural issues, and communication and media, hampering the promotion of TTM in Thailand [2]. Thus, Putiyanan and Winijkul [25] and Senachai [2] noted that the marketing strategies for the promotion of the finished forms of TTM need to be considered. Communication for health promotion is a planned process, and its effectiveness is derived when the audience has achieved, acted on, or responded to a message [26]. Although the process of marketing for medicines is very difficult process to carry out, the finished forms of TTM need to have special marketing strategies to encourage their sale among Thai consumers. Furthermore, building mutual referral systems is required [27]. Collectively, these actions may increase the uptake of the finished forms of TTM in Thailand.

### 2.3. Knowledge, Attitude, Practice (KAP), and Diffusion of Innovation (DoI) Theory

Qualitative healthcare research has been criticized for its misguided approach of separating method from theory and techniques related to conceptual underpinnings [28]. This study uses Knowledge, Attitude, Practice (KAP), and Diffusion of Innovation (DoI) theories in its conceptual framework. The KAP model is called a learning hierarchy model [29]. Its sequence proposes that, when people gain more knowledge about a certain topic, their attitude becomes more favourable, resulting in positive actions [30]. Therefore, it assumes that all of its three pillars are intertwined. However, some researchers (e.g., Launiala, 2009 [31]) argue that in certain KAP sequences, attitude and practices are not correlated. While, some studies (e.g., Wan et al., 2016 [30]) note that knowledge and attitudes can overlap.

KAP is a well-established methodology utilized in the investigation of health behaviours. It is widely employed to gather information that can help in the planning of public health programs across many countries. The national KAP studies aim to establish reliable data that can be used to understand behaviours and practices of consumers. These data can provide better information for the implementation of a national program [31]. Thus, KAP has been largely utilized in research for at least the past four decades [29].

In healthcare, K (Knowledge) stands for the understanding of health problems among people [32]. A (Attitude) refers to people’s attitudes or beliefs toward their health problems [30]. Finally, P (Practice) represents the actions based on knowledge and attitude [32]. The literature suggests that resolving individuals’ misunderstanding regarding health issues may change their attitudes and subsequent actions.

KAP studies are often referred to as the Diffusion of Innovation (DoI) framework [33,34]. By the 1950’s, diffusion researchers had begun to use the DoI framework. It applies the collective knowledge, which has been collected through naturalistic diffusion, in the testing of process interventions, to affect the spread of innovations [35]. DoI Research is a particular type of communication research [36]. The DoI studies involve mass and interpersonal communication [37], which can be divided into the sub-areas of mass media, personal communication, and Internet communication [7]. The main function of mass media communication, in the diffusion process is to create awareness about the innovation. However, the DoI field emphasizes interpersonal communication networks over any other type of communication research [33,37].

The concept of the DoI theory can be divided into two parts: “*Innovation*” and “*Diffusion*” [38,39]. When an innovation is ready to be implemented, individuals engage in the process of actively seeking information, as they want to know more about the innovation [33]. Therefore, the factors that increase the organisational capacity are considered to be of utmost importance in the implementation stage [40]. All organisations involved in healthcare are devoted to either promoting innovations or to preventing innovations from creating disruptions. Thus, healthcare professionals prioritize dynamicity in accordance with the new ideas that are spread throughout the healthcare system [41]. At the individual level, adopting health behaviour usually entails a change in the lifestyle [42]. At the organisational level, it may entail starting programs, changing regulations, or altering personnel roles. At the community level, diffusion can include using the media, advancing policies, and/or starting initiatives [43].

In Thailand’s history, TTM was neglected for over 60 years [20] and is being revived after the introduction of the 4th National Economic and Social Development Plan (1977–1981) [44]. Based on the precepts of Rogers, “the finished forms of TTM products” are considered as “the innovation” in this study. They represent the new idea of practicing, which was revived in the new environment of Thailand’s public healthcare system. The finished forms of TTM products are the object perceived as the new form of TTM by an individual or another unit of adoption that is measured by the lapse of time since its first use or discovery. Secondly, the newness of the finished forms of TTM does not involve just the new knowledge. This means that, despite the Thai people being aware of TTM for some time, they may not have developed a favourable or unfavourable attitude toward it, nor may they have adopted or rejected it entirely. Lastly, the finished forms of TTM may be desirable for one adopter in one situation, but undesirable for another potential adopter in a different situation [45]. As a result, by understanding the current trends of the finished forms of TTM and its benefits, researchers can position it effectively, maximizing its appeal [46].

In this study, KAP and DoI offered the following five valuable insights into the process of social change: (i) the qualities that enable an innovation to spread, (ii) the importance of peer–peer conversations and networks, (iii) understanding the needs of different user segments [47], (iv) identifying various health issues and potential solutions, and (v) KAP and DoI offer a component for communicating health information and activities [48].

Therefore, if practitioners in the fields of Public Health and Health Promotion want to efficiently use their resources, they must intend to reach, adopt, implement, and maintain various programs [43]. Particularly, if a health education program is viewed as an innovation, this theory could be used to describe the patterns followed by the target population seeking to adopt the program [46].

## 3. Method

In the past, various health communication programs have negated oral communication as a form of cultural production [49]. For this study, systematic procedures and tools were used to accomplish cultural production. We utilized focus group discussions with thirty experienced consumers, regarding the finished forms of TTM products in North-eastern Thailand, a part of the country that uses TTM most frequently [2,20]. The criterion, which was used to separate the groups into the primary segmentation, included the experience of having used the finished forms of TTM. Senachai [2] suggested that TTM users should be distinguished as follows: (i) individuals who have used TTM 4 times or more/year; and (ii) individuals who had used TTM 0–3 times/year (non-TTM users) (see Appendix A). 

Age range was used for the secondary segmentation is an age range. Based on a review of the literature, the consumers who had used TTM most frequently were individuals within the age range of 39–50 years. The consumers who had used it the least belonged to the 27–38 age range. Meanwhile, sex was not considered as it does not correlate to the use of traditional medicine [50,51]. As a result, the focus groups comprised three sub-groups, with the overall age range being 27–50 years. The surveyed individuals belonged to different occupations, educational levels, and had varying experiences with using finished forms of TTM (see Table 1).

The data were collected using focus groups, which were held during September 2021 via Zoom meetings. The duration of the discussion was approximately 1.5–2 h, and semi-structured interviews were utilized. A thematic analysis was adopted for the interview data, as outlined by Braun and Clarke [52]. All interviews were translated verbatim, immediately after the discussions, to avoid loss of clarification. The data transcripts were re-read and double-checked against the original audio recordings, to generate accurate Initial Codes. ATLAS.ti 22 was used to analyse the data that aligned with the study’s research aims. Analysing and coding were accomplished line by line, and almost every line in the interview transcripts was labelled. This stage was carried out to identify the initial phenomena and categorise the responses of the informants and their opinions about the finished forms of TTM. The specific questions that were asked delved into the concepts based on the KAP and DoI framework. In this step, four themes emerged from the initial codes. The first three themes were divided on the basis of the three pillars of the KAP model (Theme one: knowledge, Theme two: attitude, Theme three: practice). The fourth theme was based on the communication and promotion of the finished forms of TTM. 

The knowledge theme has various sub-themes, for example, knowledge about the efficacy of the finished forms of TTM. The attitude theme also included sub-themes such as the attitude toward the products (production, packaging, etc.) and the convenience of using them, etc. The sub-themes of the practice theme included questions such as: how do the individuals adopt the finished forms of TTM in their healthcare practice, and so on. The last theme, the communication for the promotion of the finished forms, had a sub-theme related to media, content, activities, etc., to reach the target groups.

The last step included the search for significant themes. When the data were initially coded and collated, all initial codes relevant to the research objectives were incorporated and grouped into an abstract, which focused on the analysis of the broader themes. The first three themes were re-grouped into one theme, and renamed with concise and attractive names [52]. The first theme was titled ‘The current KAP of Thai consumers toward the finished forms of TTM’, with respect to the research aims. Meanwhile, all four main themes—related to the factors for utilisation or rejection of the finished forms of TTM were reviewed and re-grouped into one theme named ‘Factors influencing the use of finished forms of TTM’. It is within the scope of the concept and adheres to the research aims. Lastly, the fourth or the communication theme was also renamed to ‘Integrated Marketing Communication (IMC) should be used as a promotion strategy to rapidly disseminate the finished forms of TTM’. It suggests the proper communication strategy to promote the finished forms of TTM. The complementary tools of ATLAS.ti 22 software and manual methods were used to select the quotes expressing the participants’ shared experiences and perceptions. Thus, these themes having contributed to the study, proving the reliability and validity of our research.

With respect to Thailand’s large population, the findings of this study are limited and cannot be generalized. Therefore, the findings herein may not reflect the experiences and opinions of all Thai consumers.

## 4. Findings

Through thematic analysis, the following three key themes were identified: (i) the current KAP of Thai consumers toward the finished forms of TTM; (ii) factors influencing the use of finished forms of TTM; and (iii) realization of integrated marketing communication as a promotion strategy to rapidly disseminate the finished forms of TTM.

### 4.1. Theme One: The Current KAP of Thais Consumers toward the Finished Forms of TTM

At the beginning of the discussion, the informants were asked to talk about their knowledge and perceptions about the finished forms of TTM. It was found that the majority of the informants had been aware of the new forms of TTM, which contrasted the results of Senachai [2]. The results indicated that, as a result of the COVID-19 pandemic, Thai consumers had achieved a significantly greater awareness about the finished forms of TTM. Particularly, some of the informants of group 2, which consisted users and non-users ranging from 35–42 years old, mentioned that that they came to know about the finished forms of The Creat and Kaempfer through television. These TTMs can be used for COVID-19 prevention, and had subsequently become popular for their form and efficacy. One of the informants commented that:

“*I actually prefer Western medicine. I always have Paracetamol at home (and) also Vitamin C, too. Nowadays, I bought the finished forms of The Creat and Kaempfer because of COVID-19. One day, I watched the television and I heard that The Creat and Kaempfer can prevent COVID-19 or (provide) treatment when we have COVID-19. So, I decided to buy both of them*.”

*(Informant 16)*

Despite not knowing whether they had received correct or incorrect knowledge, the informants decided to buy The Creat and Kaempfer. The majority of them claimed that they had felt confident, safe, and at peace when they had kept The Creat and Kaempfer in their homes, even if they felt that they did not have adequate knowledge on how to use them. This proof demonstrates the interrelationship between healthcare and psychological beliefs. Meanwhile, this study found that the informants had shifted their roles from users to advocates by instructing their family members and friends about the benefits of the finished forms of The Creat and Kaempfer.

Based on the informant’s responses, the findings highlighted the advantages of the new form over the traditional form (Medicinal pots). In fact, some informants claimed that the finished forms of TTM had been extracted and capsulized. Thus, they had expressed a higher level of trust in the quality, cleanliness, and packaging of the finished forms. Consequently, it was determined that the majority of the informants would use some of the finished forms of TTM in the future as replacements to some modern medicines in their healthcare. Concisely, this study was able to confirm that the majority of informants had been able to recognize the advantages of the finished forms of TTM. They had accomplished this by relating the finished forms of TTM to its traditional forms and the COVID-19 virus. As one informant stated:

“*The finished forms of TTM are packed in the box with label and description like conventional medicines. I think they are really good. Most people will buy them because they look modern and clean. I usually buy the turmeric at home but nowadays I bought the Kaempfer during COVID-19*.”

*(Informant 10)*

However, some negative viewpoints were also expressed with respect to the production process of the finished forms of TTM and the products’ packaging and design. The following conditions were found to exist: (i) the products are underdeveloped, (ii) the products look cheap, (iii) the products have not been registered, and (iv) counterfeit TTM. One-third of the informants strongly stated that the packaging of most of the finished forms of TTM looked old and cheap. In many cases, the labels had faded and could no longer be read. Some of the informants mentioned that they had been unable to see the expiration dates and the food and drug guarantee. This included counterfeit TTMs, which had entered the market during the pre-COVID period and the COVID-19 pandemic. Particularly, they included counterfeit forms of The Creat and Kaempfer. Moreover, the prices had increased 2–5 times the original price, becoming more expensive as the demand increased. Thus, the pandemic caused the finished forms of TTM to become overpriced. Nonetheless, the findings indicated that products with colourful packaging were being easily sold compared to products in plain coloured packaging, as people thought the latter seemed untrustworthy. For instance, one informant noted that:

“*I have seen some good-quality finished forms of TTM, but some are still underdeveloped. These days Thais want the Creat to prevent COVID-19; it is even worse because fake products are more on the market. The vendors should not do like this because it is the health of the people*.”

*(Informant 23)*

Similar to other research studies, the major problem related to the finished forms of TTM is the knowledge of the users regarding traditional versus Western medicine. Therefore, the conflict in knowledge and attitude toward the use of the finished forms of TTM still pertains. For instance, a non-user informant (informant no. 8) mentioned that: “*I don’t know what kind of finished forms of TTM I should take if I have (a) fever*. *I know only Paracetamol so I will take them if I have fever or headache*.” Some informants strongly noted that, despite the slower recovery, some finished forms of TTM can prevent diseases that cannot be healed using Western medicines, such as cancer and COVID-19. This statement was similar to those in Senachai [2], in which it was noted that incorrect knowledge and beliefs about traditional treatments and medicines still exist. Finally, the informants suggested that despite the efficacy of the finished forms of TTM, there exist serious concerns about their image and reputation. 

### 4.2. Theme Two: Factors Influencing the Use of Finished Forms of TTM

It was found that the usage of the finished forms of TTM’s depended upon the following factors:

(1) Consumer’s knowledge and beliefs; certain personal interest in the finished forms of TTM is related to positive actions.
(2) Accessibility and convenience for consumers; it was noted that the usage of finished forms also depends on the medicines that the consumers have at home and the finished forms of TTM that are widely available in the marketplace.
(3) Product quality; issues about the quality and efficacy (a slower recovery rate and academic proof) of the finished forms, and the product design. When the consumers decide whether or not they want to use the finished forms of TTM, product quality is significantly considered, particularly, in the case of middle class and well-educated consumers.
(4) Promotion of the finished forms of TTM and drug advertisements.
(5) The pandemic; it is one of the major factors that has encouraged the uptake of the finished forms of TTM, as the informants educated themselves about COVID-19 by watching television. One informant stated that:
  “*Finished herbal medicine makes us more convenience because it easy to take than the old form. I can easily find them from drug stores too*.”
  *(Informant 13)*

To further promote the use of finished forms of TTM, experienced consumers strongly stated that the producers must focus on two areas: research and product design. Further research is required to support the efficacy of the finished forms, and better product design must be achieved before the products are sold in the marketplace. As a result, they have urged the government to help the vendors in developing high-quality TTM products, to regain the trust of the Thai people. This can be accomplished by emphasising the safety of the TTM products, which should include better packaging and suitability for each target group, particularly, the middle class and the well-educated. It should also consider social status related to health and well-being of people.

The informants primarily mentioned that, to cater to the target groups, the promoters should focus on the frequent users of the finished forms of TTM (e.g., mothers). Hypothetically, encouraging mothers to use the finished forms of TTM to treat younger children may help in spreading accurate information about the finished forms of TTM. It may also help the children to adopt the use of TTM. Some informants strongly noted that the MoPH should promote the usage of the finished forms of TTM to treat minor illnesses, such as fevers, headaches, and stomach aches. This would help Thai citizens to manage their own health, thereby allowing the government to save on healthcare costs and promote the well-being of the people. This evidence indicates that there is a significant relationship between caregivers and the use of the finished forms of TTM. Moreover, in Thai culture, healthcare is a part of the larger social system, which involves women, families, communities, the society, and the economy. Therefore, social factors, health, and the economy, are inevitably intertwined, as noted by one informant stating that:

“*When I have a fever, I had better use The Creat. I also use Turmeric when stomach aches, which is more compatible for me. The government should promote both of them because they are good for our health*.”

*(Informant 21)*

Many informants also mentioned that the MoPH should avail of the opportunities afforded by the COVID-19 pandemic, and promote the usefulness of herbs and finished forms of TTM. This is because many people have become aware of the finished forms of TTM, more than they were before. They also claimed that the trialability or testability of the finished forms is acquired by providing good examples of the effectivity of TTM. This can help people to better understand the concept of TTM and its efficacy. Thus, there may be an increase in the rate of adoption of the finished forms of TTM.

According to the strategy and key statements expressed by the informants, to encourage Thai people to adopt the finished forms of TTM, the following measures can be opted:

- The promoters should encourage the usage of the finished forms of TTM by primarily dispensing information to caregivers about the five most well-known herbs in their finished forms, which can be used to replace modern medicines for minor illnesses. The informants specifically noted that, instead of consuming The Creat and Kaempfer during COVID-19 times or for curing stomach aches, sore throats, and diabetes, they preferred using herbal products.
- Regarding the information about the properties of herbs/the finished forms of TTM, research should be carried out on the topic of drug production. Moreover, this research should be conducted to support the efficacy of TTM and foster a greater sense of trust toward TTM among the people, particularly the well-educated class.
- The advantages of using the finished forms of TTM should be communicated positively without negating the usage of modern medicines. Some information about the disadvantages of using the finished forms of TTM should also be shared with the public. For instance, one informant said that:
  “*If we want to promote our medicines, we should make it clear about their advantages. Anyway, it would be best if you do not say that they are better than western medicines because people will be against them. Just focus on the good points of traditional medicines that have been changed from the old form people will be ok with that*.”
  *(Informant 30)*

### 4.3. Theme Three: Integrated Marketing Communication (IMC) Should Be Used as a Strategy to Rapidly Disseminate the Finished Forms of TTM

The statements from the majority of the informants clearly noted that many types of media should be used to inform the public, particularly people with modern beliefs and teenagers. Subsequently, each informant was urged to rate the top five types of media that they felt were best suited to promote the finished forms of TTM. The findings indicate that television is the most popular form of media in disseminating information about TTMs, followed by YouTube, social media, personal media, and radio, respectively. However, with the exception of print media, most informants, regardless of their age, stated that promoters should use various types of media. The print media were excluded because, according to the informants, most Thai citizens are illiterate and/or uninterested in reading. Hypothetically, integrated marketing communication (IMC) is well-suited to promote finished forms of TTM in the marketplace.

The informants were then encouraged to create suitable promotion campaigns. The results are summarized as follows.

#### 4.3.1. Television Advertising about the Finished Forms of TTM across the Country Should Be Conducted

Regarding television media, many informants frequently mentioned that promoters should mainly promote and disseminate information about the finished forms of TTM on television. This is because all members of their family watch television. Once people have seen the news and received the accurate information about the finished forms of TTM, it is possible that three events will occur. First, people will remember that the finished forms of TTM are available. Second, people will become engaged, and try to consume one or more finished forms of TTM. These products will include traditional TTMs that people are already aware about, such as Turmeric, The Creat, and Kaempfer. Third, when people visit the pharmacy, they may ask for the finished forms of TTM. Furthermore, it was indicated that promoters should broadcast public service announcements, which would allow large-scale dissemination of the information related to the finished forms of TTM. 

The advertising segments on television should promote the positive aspects of the finished forms of TTM without negating the usage of modern medicines. This evidence indicated that the informants continued to favour Western medicines, and were strongly engaged with them, having adopted them as a part of their healthcare practices. Specifically, the most suitable media of reaching certain demographics were indicated as follows: (a) to reach teenagers, advertising should be broadcasted during sports or entertainment programming and (b) to appeal to mothers and the elderly, advertising should be broadcast during the news and the drama programming. Furthermore, the informants suggested that these advertisements should not be over one minute in length. Instead, they should be re-broadcast multiple times a day.

The act of using finished forms of TTM needs to be inserted into the storylines of soap operas. A few of the respondents shared their experience of discovering herbal medicine through Thai soap operas on television. This will help mothers and the elderly in their awareness about the finished forms of TTM.

When researchers discover new information about herbs and their properties, it should be shared with the public. This should include the innovation of the finished forms of TTM that have undergone the transformation from raw herbs and medicinal pots to other new forms (finished forms). The development of modern traditional medicines could be demonstrated to the Thai people, as these medicines are easier to consume and more convenient to buy compared to the traditional forms. Repeatedly, this statement shows that convenience of use is a major factor that determines whether consumers choose to adopt or reject TTM. For example, one informant reported that:

“*The program on TV must get attention and a short statement to impress the audiences. People who are creating the content have to be trustworthy in the topic of creation in TV. Personally, I like they insert the topic related to our traditional medicines in soap operas. I think it is more interesting than any program.*”

*(Informant 22)*

#### 4.3.2. Using YouTube Posting on Social Media for Teenagers

Regarding YouTube, a large number of informants agreed that it is possible to gain access to teenagers through YouTube. Accordingly, if teenagers have to draft educational reports about herbs and medicines and related subjects, the media content that focuses on this area is also important. Providing information that can be useful to teenagers through platforms such as YouTube may be effective in disseminating information about finished forms of TTM. YouTube is especially effective because of the following reasons: (1) it is a fast communications channel, (2) videos or illustrations can be easily created to advance knowledge about the finished forms of TTM, and (3) to attract people’s attention, the information can be quickly uploaded to the Internet. Moreover, majority of the informants noted that YouTube videos should be posted on Facebook because many Thai people are significantly engaged in using Facebook, more than in other forms of social media.

#### 4.3.3. Personal Media to Approach the Rural People

The informants mentioned that personal media should also be used to dispense information about TTMs to fellow Thai citizens. The most frequently mentioned individuals were public health volunteers, as they helped the doctors during the spread of the COVID-19 pandemic in Thailand. Accordingly, the role of the public health volunteers was to visit the villagers in their areas. Being familiar with the villagers, they would be able to visit them at their homes. The informants noted that, when conditions in Thailand return to normal, the MoPH should assign public health volunteers to visit the rural areas and disseminate information about the finished forms of TTM to the villagers. See comment below:

“*Since we have COVID-19, public health volunteers always come to our house to check if we are ok. I think the promoters should train them and provide some documents so that they can educate us.*”

*(Informant 20)*

Specific to the information that the informants wanted the MoPH and promoters to provide was the properties of the finished forms of TTM including the following actions: (1) sharing information about the finished forms of TTM that are more effective than Western medicines, and (2) demonstrating the use of the actual TTMs to assist people in becoming more aware of the new finished forms. They also noted that the new finished forms of TTM such as Turmeric and The Creat should become well-known. This is because Thai citizens have known about them since they were used as raw herbs in medicinal pots and are now available in new forms. Thus, they may accept and adopt these forms more readily. Hypothetically, the innovation must relate to the compatibility of the people’s existing knowledge. 

#### 4.3.4. Public Relations about the Finished Forms of TTM by Using Radio and Local Community Broadcast

A few informants stated that radio/local community radio would be effective in disseminating information to the villagers in remote areas. They also noted that it would be particularly effective to reach the elderly, as they comprise the majority of radio listeners. Moreover, it would help people in learning more about the finished forms of TTM. 

With respect to radio programs, there should be programs that feature discussions about the properties and usefulness of herbs. In fact, informant 25 stated the following: “*We should know about the many ways to use herbs, which is not only for (the) treatment (of) humans, but also (for the) treatment (of) the animals and using for agriculture*.” They also noted that such radio/community radio programs should be broadcasted three times a day for 1–3 min. This approach may help to promote the finished forms of TTM to rural people.

In summary, to reach every demographic of Thai consumers, below-the-line and above-the-line advertising strategies should be implemented. Therefore, the products should be properly promoted and demonstrated, allowing the consumers to personally investigate and consume such products using a high-touch experience. Meanwhile, above-the-line advertising is best suited for boosting large-scale awareness of the finished forms of TTM. This is because advertising has an impressive impact on consumers’ decision-making and recognition.

## 5. Discussion and Conclusions

The revival of TTM’s policy occurred in the new environment of Thailand’s public healthcare system and represents new ideas of healthcare practice [7]. Clearly, governmental policies and financial support have been the driving forces of TTM services and their dissemination to the public. They have also helped to increase the people’s awareness about its role in health promotion [20]. Senachai [2] showed that the MoPH is widely promoting the use of the finished forms of TTM, predominantly targeting self-care at the family level [7]. In Thailand, one common reason to use traditional treatments and medicines is its alignment with sociocultural, religious, spiritual values [20,21,27], and cuisine [21]. A study conducted by expert researchers Tan, Otake, Tamming, Akuredusenge, Uwinama, and Hagenimana [27], in Rwanda, noted that the characteristics of traditional medicines are to respond to the community members’ health, along with their social and economic needs. Similarly, our findings confirmed that individual belief, social, economic and communication factors are intertwined with healthcare practices. 

Presently, Thailand is supporting the finished forms of TTM products to ensure their quality, efficacy, and safety. It is also trying to improve the trustworthiness about their usage by consistently and systematically enhancing the competitive advantage of TTMs’ reputation [53]. Accordingly, KAP and DoI offer valuable insights into the process of social change by identifying the qualities needed by an innovation to be adopted. When compared with traditional ideas, innovations should be advantageous, cost-effective, time-saving, and compatible with legacy knowledge [37,38]. Furthermore, they should have lower complexity, along with easy testability and observability [38]. As a result, these innovations will be adopted faster than others [54]. 

The findings indicate that knowledge and attitude toward the innovation of new finished forms of TTM can overlap, resulting in the KAP consequence that occurred in Wan, Rav-Marathe, and Marathe [30]. When the consumer participants are aware of the innovation of new finished forms of TTM, their knowledge and attitude about the product quality and its accessibility increases. The advertisement broadcasted during the COVID-19 pandemic encouraged consumers to use new forms of TTM. Accordingly, the innovation of the finished forms of TTM showed a relative advantage over Western medicines. The consumers have perceived the new forms to be better than the traditional form. Additionally, the finished forms are perceived to be more suitable to the lifestyles of the intended consumers, compared to the current practices. This is because traditional forms are more meaningful considering the prevalent values and norms of the Thai social system. These two characteristics are demonstrating the competitive advantage of TTMs over Western medicines, which is why Thai consumers should develop positive attitudes toward the finished forms of TTMs. This study also noted that the degree of relative advantages of the new forms of TTM (finished forms) are related to social-prestige, economics, convenience, and the satisfaction of the consumers, with respect to product quality. Given the social contexts, which include mistrust and insecurity, our findings suggest that complex situations, such as the COVID-19 pandemic, may have emphasised the issues related to interpersonal and community relationships. This includes the use of mass media and news events broadcasted during the COVID-19 pandemic. These media can be utilized to increase the visibility and the prominence of the finished forms of TTM [38].

Unfortunately, the key failures in promoting the finished forms of TTM originate from the biases and bad attitudes of people, who have been influenced by Western beliefs. Thus, the lack of knowledge pertaining to the use the finished forms of TTM represents an obstacle. This hurdle is one of the main issues faced by the social system of Thailand, when individuals try to use the finished forms of TTM. Accordingly, the complexity of the usage of finished forms of TTM is negatively correlated with the rate of adoption of the finished forms of TTM. This study discovered that the complexity of usage of the finished forms of TTM is related to the knowledge of how to use them and where to buy them, which has resulted in lesser trial ability and adoption. Although, there is a need for more knowledge in this aspect, support from prior adopters or other sources can increase the chances of adoption [55]. However, the perceptions, convenience of use, and effectiveness of the finished forms of TTM were found to be insufficient compared to the influence of Western medicines. This also included the packaging of the finished forms of TTM, which seemed ‘cheap’ or ‘old’. Thus, packaging was also found to strongly influence the consumers’ purchasing decisions regarding the finished products, especially among middle-class people [2].

Many researchers in Thailand (e.g., Putiyanan & Winijkul, 2008 [25]) have claimed that TMs’ product quality, packaging, and design of the packaging looked cheap and unclean. Additionally, unclear expiration dates or absence of expiration dates on the finished products also posed an issue. Moreover, some products did not meet international standards, leading them to be perceived as counterfeit [56]. All factors reflected poorly on the reliability of the products. Tan, Otake, Tamming, Akuredusenge, Uwinama, and Hagenimana [27] noted that herbal medicines are always perceived to have certain safety issues, such as unclear expiration dates and/or vague instructions. This can result in overdosing or other harmful consequences. Kim [57] reported that incorrect information will undermine consumers’ trust and their assessments of health information.

### 5.1. Practical Implications

Health communication takes a top-down approach [18]. However, the MoPH characteristics must match the individual Thai characteristics to encourage the adoption of finished forms of TTM in Thailand. This means that the MoPH must have an intention to change (motivation and ability), an innovation-system fit (compatibility), an assessment of implications (observability), and a political mandate [58]. The success of the diffusion and the adoption of the finished forms of TTM requires the involvement of the MoPH and its agents at an exclusive level. They must be empowered to work together, solve problems and exercise effective communication through various engagement activities and media. This would allow the transformation of information into knowledge and actions that would foster a positive attitude among Thai citizens. This includes supporting promoters and helping them to continuously develop their marketing mix and public relations activities, thereby creating positive attitudes toward the finished forms of TTM. These actions only will lead to the enhancement of consumer satisfaction [59].

According to Rogers, a communication channel is a process in which participants can create and share information to reach a mutual understanding [37,45]. The information diffusion process typically involves mass media and interpersonal communication channels [37], practicing the following activities: (1) social marketing techniques [38,60], (2) the assessment of IMC [60], and (3) social networking through communication channels that can rapidly disseminate information in the social system [38].

To encourage the usage of the finished forms of TTM, we analyse the findings of our study to suggest that social marketing and IMC are extremely important in disseminating information. This is because these media can enhance consumer knowledge and foster trust among Thai consumers, promoting the finished forms of TTM. Moreover, this would lead to campaign development, which is more consistent. Accordingly, social marketing has proven to be successful in achieving sustained behavioural changes in a diverse range of health-related behaviours [61]. Meanwhile, IMC can play a vital role in health-related communication [60]. Thus, the government should pay great importance to these media of communication, to promote the finished forms of TTM among consumers. Especially, media, content, and related activities should be employed to target various demographics and their respective behaviours.

### 5.2. Recommendations and Suggestions for Health-Related Officers

The IMC promotions mainly focus on communication, awareness, knowledge, liking, preference, conviction, and behaviours. This is often called the hierarchy of effects model, which maps out the response process that the receiver of a message undergoes before performing actual actions [62]. The communication and media campaign about the finished forms of TTM is related to the hierarchy of effects model. Thus, this study proposes that the Thai government should implement the following measures: 

(1) The awareness and knowledge about finished forms of TTMs should be encouraged by using evidence to provide the correct information. This could prove the efficacy of the finished forms of TTM. It is important to utilize mass media and news channels to increase the visibility and the prominence of the finished forms of TTM. This is because the main function of mass media and communication is to create awareness and knowledge about innovations [63]. Key and Czaplewski [62] noted that advertising is an excellent tool for raising awareness and conveying knowledge about a social issue. The results and the consumers suggest that ‘The Creat’ (or other finished forms of TTM) should be promoted using televised advertisements as they have a greater impact on consumers compared to other forms of media.

Additionally, it has been suggested that public health institutions should focus on social media activities to achieve the goal of health communication [64], because social media can create sustainable networking and interactive participation [65]. The findings confirmed that utilizing YouTube to disseminate information about the finished forms of TTM on social media (Facebook) can help in reaching teenagers. This will create an understanding about the finished forms of TTM in the younger population. Wu and Feng [65] reported that young adults can communicate and gain more information through interactions with people regarding health-related social media, improving their health awareness. 

The information about the properties of the finished forms of TTM and their advantages can be communicated through public service announcements on the radio/community radio. This is important in reaching the rural and elderly population. By communicating IMC messages to the Thai populace, proper knowledge and consistent information about the finished forms of TTM may be promoted, developed and shared. Over time, this information may be configured and assimilated within the contexts of Thai culture, structure, and environment [2].

To accelerate the adoption process, it is crucial to understand the requirements of different user segments [47]. Specifically, this study noted that the promotion of the finished forms of TTM must employ a unique proposition focusing on the customers and market. Instead of promoting various finished forms at once, the government promoters should specifically promote the top five leading finished forms of TTM products. Additionally, the MoPH and the promoters should encourage consumers to use the finished forms of TTM to prevent or cure minor illness such as fevers, headaches, and stomach aches, that are common among Thai citizens. This study also noted that, in promoting the use of the finished forms of TTM, mothers are vital individuals. The MoPH should pay special attention to mothers and caregivers as they have a significant relationship with the use of traditional medicines, especially using traditional medicines for children [66]. Accordingly, it will be possible to bridge the knowledge gap between traditional treatments and medicines [67]. Consequently, if mass media are used to promote the leading finished forms of TTM products, detailed insights are employed to reach target audiences, the right channels of promotion are selected, and effective messaging is practiced, then Thai consumers can obtain the correct knowledge pertaining to TTMs. 

(2) After the consumers have been provided with the accurate knowledge, this study noted the need for a change in consumers’ likings, preferences, and attitudes toward the finished forms of TTM’s. More specifically, the reputation of TTMs should be enhanced Improvements such as suitable and clean packaging would be best suited to enhance TTMs’ reputation across all target groups. Putiyanan and Winijkul [25] also reported that the attitudes of their sample group became more positive when the packaging of the finished forms of TTM was improved. Thus, such enhancements can assist consumers in cultivating a more positive attitude toward the products. Similarly, Senachai [2] also claimed that since the finished forms of TTM have been extracted, capsulized, and placed in cleaner packaging, it has been easier for consumers to accept and use them. The current findings also confirm that packaging and cleanliness can help to increase the acceptance of finished forms of the TTM product. This is because the consumers can read the label and obtain more information about the product before they made their purchasing decisions.

This study also noted that the promoters should continuously use advertising to promote the finished forms of TTM. The methods of advertising should vary and must inculcate TTM usage (both traditional and finished forms) into the storylines of television soap operas. Moreover, documentaries focusing on TTM should be created and broadcasted. Furthermore, public relations are an excellent tool for building affinity and preference [62]. The MoPH must deploy clear and correct information to inform the public about the finished forms of TTM. Most importantly, the MoPH should widely promote the benefits of the finished forms of TTM to restore trust among the Thai population and continue to build on that trust. 

(3) To affect behaviours and promote consumer conviction, the MoPH must encourage consumers to develop real experiences with the usage of the finished forms of TTM. In this stage, the importance of interpersonal contacts in the diffusion of information relies upon ‘*Who talks to whom*’ [68]. Clearly, personal marketing is best to convert preferences and conviction into behaviours [62]. This is because promoting changes in behaviours is often related to peer influence, peer education, or peer networks [68]. Consequently, personal media can facilitate the consumers’ decisions to adopt the finished forms of TTM by implementing the appropriate tactics. Accordingly, this study proposes that public health volunteers can be used as communicators for interpersonal/two-way communication. This would allow for the successful dissemination of information across various communities, as Thai citizens rely on personal media, particularly physicians and the staff members of the Public Health Service for data regarding medications and healthcare [25,59].

Lastly, the experiences (testimonials) of individuals who have successfully used the finished forms of TTM should be employed to foster consumers’ decision-making processes. This would enable them to adopt the finished forms of TTM. The results noted that consumers should share health-related information with their family members and friends especially if they had positive experiences using finished forms of TTM. Senachai (2019) reported that, when medical doctors had applied their knowledge and experience of using traditional treatments and medicines to their professional practices in hospitals or clinics, they had received positive results. In fact, both medical doctors and consumers are engaged in the same process of acceptance, which pays particular attention to the efficiency of the treatment [69]. Consequently, this study has claimed that we can reach the confirmation stage by using experiences to create customer advocacy. It can help to create permanent behaviours, which would empower consumers to continue to use the finished forms of TTM. Over time, such consumers would become permanent users. Given this fact, it is imperative to craft a new image and reputation for the finished products of TTMs. This image should enhance the portrayal of the safety, reliability, and efficacy of the finished forms of TTM. This can be achieved through effective communication and promotion, which can further accelerate the rate of diffusion of the finished forms of TTM within the marketplaces of Thailand.

This study has contributed to the body of literature related to traditional Thai medicine; however, limitations are inevitable. This study utilised focus groups from Thailand; therefore, the outcomes might be substantially limited and cannot be generalised to the greater Thai population. Further research is recommended through the conduct of qualitative in-depth interviews and/or quantitative experimental studies that test the efficiency of variance.

## Figures and Tables

**Table 1:** Summary of informants.

Group	Age Range	Users	Non-Users	Total
1	27–34 years old	5	5	10
2	35–42 years old	5	5	10
3	43–50 years old	5	5	10
**Total**		15	15	30

## Data Availability

Available upon request.

## Appendix A

**Table A1:** The Total Number of Informants Located in Khon Kaen Province Per Group.

Informants	Group	Gender	Age Range	Education	Occupation	Experience
Informant 1	1	F	27–34	Bachelor’s degree	Government staff	User
Informant 2	1	F	27–34	Bachelor’s degree	Homemaker	User
Informant 3	1	F	27–34	Bachelor’s degree	Self-employed	User
Informant 4	1	M	27–34	Vocational College	Homemaker	User
Informant 5	1	M	27–34	Senior High School	Self-employed	User
Informant 6	1	F	27–34	Vocational College	Government staff	Non-user
Informant 7	1	M	27–34	Bachelor’s degree	Employee	Non-user
Informant 8	1	M	27–34	Senior High School	Self-employed	Non-user
Informant 9	1	M	27–34	Bachelor’s degree	Employee	Non-user
Informant 10	1	F	27–34	Senior High School	Homemaker	Non-user
Informant 11	2	F	35–42	Bachelor’s degree	Government staff	User
Informant 12	2	M	35–42	Senior High School	Homemaker	User
Informant 13	2	F	35–42	Bachelor’s degree	Employee	User
Informant 14	2	M	35–42	Vocational College	Self-employed	User
Informant 15	2	M	35–42	Senior High School	Self-employed	User
Informant 16	2	M	35–42	Bachelor’s degree	Self-employed	Non-user
Informant 17	2	F	35–42	Senior High School	Employee	Non-user
Informant 18	2	F	35–42	Senior High School	Farmer	Non-user
Informant 19	2	F	35–42	Bachelor’s degree	Government staff	Non-user
Informant 20	2	M	35–42	Senior High School	Farmer	Non-user
Informant 21	3	F	43–50	Bachelor’s degree	Government staff	User
Informant 22	3	F	43–50	Vocational College	Self-employed	User
Informant 23	3	M	43–50	Bachelor’s degree	Government staff	User
Informant 24	3	F	43–50	Senior High School	Homemaker	User
Informant 25	3	M	43–50	Vocational College	Employee	User
Informant 26	3	F	43–50	Bachelor’s degree	Homemaker	Non-user
Informant 27	3	F	43–50	Bachelor’s degree	Employee	Non-user
Informant 28	3	M	43–50	Senior High School	Self-employed	Non-user
Informant 29	3	M	43–50	Vocational College	Self-employed	Non-user
Informant 30	3	M	43–50	Bachelor’s degree	Government staff	Non-user
